# Empirical Validation of a Multidirectional Ultrasonic Pedestrian Detection System for Heavy-Duty Vehicles Under Adverse Weather Conditions

**DOI:** 10.3390/s25175287

**Published:** 2025-08-25

**Authors:** Hyeon-Suk Jeong, Jong-Hoon Kim

**Affiliations:** Department of Highway & Transportation Research, Korea Institute of Civil Engineering and Building Technology, Goyang-si 10223, Republic of Korea; jhs2204@kict.re.kr

**Keywords:** ultrasonic sensor, pedestrian detection, heavy-duty vehicle, adverse weather, blind-spot monitoring

## Abstract

Pedestrian accidents involving heavy vehicles such as trucks and buses remain a critical safety issue, primarily due to structural blind spots. While existing systems like radar-based FCW and BSD have been adopted, they are not fully optimized for pedestrian detection, particularly under adverse weather conditions. This study focused on the empirical validation of a 360-degree pedestrian collision avoidance system using multichannel ultrasonic sensors specifically designed for heavy-duty vehicles. Eight sensors were strategically positioned to ensure full spatial coverage, and scenario-based field experiments were conducted under controlled rain (50 mm/h) and fog (visibility <30 m) conditions. Pedestrian detection performance was evaluated across six distance intervals (50–300 cm) using indicators such as mean absolute error (*MAE*), coefficient of variation (*CV*), and false-negative rate (*FNR*). The results demonstrated that the system maintained average accuracy of 97.5% even under adverse weather. Although rain affected near-range detection (*FNR* up to 17.5% at 100 cm), performance remained robust at mid-to-long ranges. Fog conditions led to lower variance and fewer detection failures. These empirical findings demonstrate the system’s effectiveness and robustness in real-world conditions and emphasize the importance of evaluating both distance accuracy and detection reliability in pedestrian safety applications.

## 1. Introduction

Recently, the South Korean government has been promoting the adoption of pedestrian detection systems in conjunction with stricter enforcement measures aimed at reducing pedestrian traffic accidents. Despite these efforts, the number of fatalities and injuries caused by vehicle vs. pedestrian collisions remains high. Notably, approximately 19% of pedestrian traffic accidents involve large vehicles such as trucks and buses, with most incidents attributed to blind spots in the driver’s field of view. These blind-spot issues are particularly pronounced in complex urban intersections, such as crosswalks and right-turn zones, increasing the likelihood of collisions with vulnerable road users (VRUs).

Current pedestrian detection technologies primarily utilize radar-based ADAS systems such as forward collision warning (FCW) and blind-spot detection (BSD). However, these systems are predominantly installed in high-end passenger vehicles and are not specifically optimized for pedestrian detection. As a result, there is a growing need for technological advancements aimed at eliminating blind spots to effectively prevent pedestrian accidents. While the adoption of ADAS safety technologies has been actively progressing in passenger vehicles, large commercial vehicles—such as trucks and buses—have been largely excluded from such developments. In particular, aging commercial vehicles currently in operation remain highly vulnerable to collisions at high-risk locations such as crosswalks and intersections.

Given these challenges, it is necessary to analyze the technical limitations of existing VRU detection sensors. Ultrasonic sensors offer reliable short-range, unidirectional detection (up to approximately 5 m), but are ineffective for detecting pedestrians at longer distances. ADAS radars support long-range detection (up to 250 m), but their dual-beam structure enables wide-angle detection only at close range and narrow-angle detection at longer ranges. LiDAR provides the advantage of 360-degree omnidirectional sensing with a single sensor; however, in the case of tall commercial vehicles, near-field blind spots may occur due to the vehicle’s height. Additionally, LiDAR’s low frame rate imposes limitations on detecting fast-approaching nearby objects.

The purpose of this study was to empirically demonstrate a multichannel ultrasonic sensor-based system designed for 360-degree pedestrian collision avoidance around large commercial vehicles. The system was evaluated under various environmental conditions, particularly adverse weather, through quantitative performance assessment. This research aimed to review the potential for developing a system that not only effectively covers blind spots but is also practically applicable to commercial vehicle platforms.

To this end, a multichannel ultrasonic sensor array was designed based on vehicle structure and sensor characteristics. Scenario-based experiments were conducted to reflect real-world road environments, enabling quantitative evaluation of the sensor’s detection performance. In addition to normal weather conditions, this study also analyzed pedestrian recognition performance under adverse conditions such as rain and fog, providing insights into the proposed system’s applicability in real traffic environments.

While most next-generation ADAS/AV systems employ a fusion of LiDAR, thermal imaging, and camera-based vision to enable high-level autonomy, these solutions often struggle in adverse weather conditions due to sensor degradation. For example, optical cameras and LiDAR may experience significant performance drops in heavy rain or fog, whereas ultrasonic sensors are less affected due to their acoustic nature.

Therefore, this study focused on ultrasonic sensing as a complementary layer within the broader sensor stack, particularly for near-field blind-spot detection in large vehicles. The proposed multichannel ultrasonic ring configuration is not intended to replace existing sensors, but to fill critical coverage gaps where conventional sensors perform poorly. This practical approach is especially suited for retrofitting legacy commercial fleets, where cost, complexity, and environmental robustness are key concerns.

## 2. Literature Review

### 2.1. Trends in Pedestrian Collision Avoidance Technologies

Forward collision warning (FCW) and forward collision-avoidance assist (FCA) systems are core components of modern vehicle safety technologies designed to detect and respond to hazards in the forward path of the vehicle to prevent or mitigate collisions. FCW utilizes radar sensors and cameras to detect vehicles ahead, calculate the inter-vehicle distance and relative speed, and determine the risk of collision. Upon identifying a threat, the system alerts the driver through audible and visual warnings. FCA extends the functionality of FCW by incorporating automatic emergency braking to reduce the severity of a collision or avoid it entirely. According to research by the Insurance Institute for Highway Safety (IIHS), vehicles equipped with FCW experience a 17% reduction in crash rates, while those with FCA show up to a 43% reduction. Recent FCA systems have evolved to detect not only vehicles but also pedestrians and cyclists; however, their collision avoidance performance significantly declines at speeds exceeding 55 mph.

Rear cross-traffic collision warning and assist systems (RCCW, RCW, RCCA, RCA) are essential safety technologies designed to detect and respond to hazards during vehicle reversing, thereby reducing the likelihood and impact of rear-end collisions. RCCW utilizes rear radar sensors to detect approaching vehicles from the left or right when reversing and provides the driver with audible and visual alerts. RCCA and RCA enhance RCCW by incorporating autonomous braking functionality, allowing the vehicle to stop automatically when a collision risk is detected, even without driver intervention. Advanced RCCA systems are capable of detecting not only vehicles but also pedestrians in the rear detection zone.

Blind-spot detection (BSD) systems are advanced driver-assistance technologies that monitor areas outside the driver’s direct line of sight, particularly the rear-side and rear-quarter zones, where collision risks are higher in large vehicles. These systems employ infrared sensors, radar, cameras, and AI-based object recognition algorithms to detect pedestrians, cyclists, and motorcyclists and provide visual and auditory alerts to the driver. BSD systems are increasingly being integrated with other ADAS features, evolving into comprehensive safety systems that include forward and rear cross-traffic collision prevention, lane departure warnings, and emergency braking functionalities.

### 2.2. Pedestrian Collision Safety Scenarios

Scenario-based evaluation studies aimed at preventing pedestrian collisions have become a critical methodology for quantitatively validating the performance and real-world applicability of detection systems. The Federal Transit Administration (FTA) adopted this approach in its empirical evaluation of the pedestrian avoidance safety system (PASS). Although the PASS sensor system was tested across various vehicle types, its application to large vehicles (e.g., buses) remained limited. To address this gap, the FTA conducted an alpha test on a closed test bed at the Virginia Smart Road, simulating real-world driving conditions. The experimental setup replicated a virtual intersection environment based on actual Pierce Transit bus routes and included key infrastructure elements such as lane markings, stop lines, streetlights, and bus stop waiting areas.

To simulate pedestrian activity, a computer-controlled belt system and vulnerable road user (VRU) mannequins were employed. These mannequins were programmed to traverse crosswalks at walking and running speeds. High-risk scenarios were also implemented, such as pedestrians suddenly emerging from behind parked roadside vehicles or entering crosswalks on curved roads where the driver’s line of sight is obstructed. The experiments accounted for both daytime and nighttime lighting conditions, as well as adverse weather conditions like rain and fog, thereby replicating near-realistic traffic environments. All scenarios were developed in accordance with the VRU protection protocols outlined in Euro NCAP and SAE J3029, enabling a quantitative evaluation of PASS in terms of detection accuracy and response performance.

**Figure 1:**
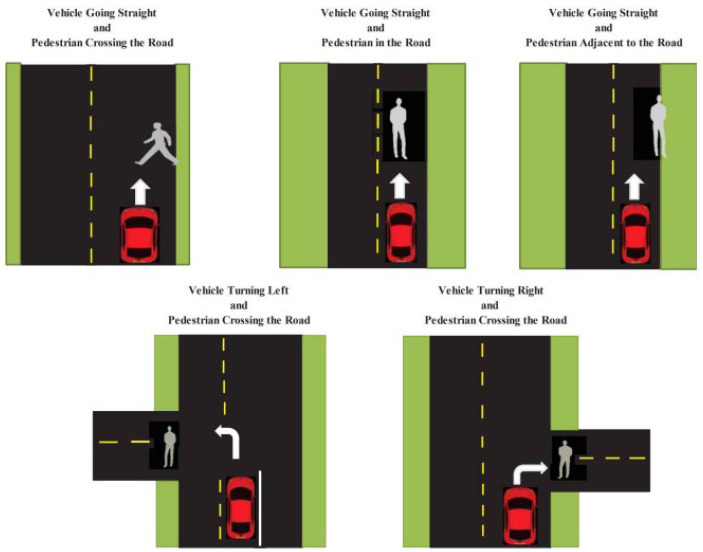
Five priority pre-crash scenarios [1].

In addition, the U.S. National Highway Traffic Safety Administration [1] defined critical pedestrian–vehicle crash scenarios by analyzing accident data to support the development of communication-based collision prevention technologies. Using data from the fatality analysis reporting system (FARS) and the general estimates system (GES), the NHTSA identified high-risk pedestrian collision patterns and proposed five priority scenarios based on accident frequency and social cost (Figure 1). These scenarios have since served as a foundational reference for the design and policy development of vehicle-to-pedestrian (V2P) systems.

Euro NCAP has proposed evaluation criteria for advanced driver assistance systems (ADASs) aimed at protecting a wide range of VRUs, including pedestrians, cyclists, and motorcyclists. To assess the effectiveness of features such as autonomous emergency braking (AEB) and lane-keeping assist systems, a variety of traffic and driving scenarios were developed. The test conditions were set within a temperature range of 5–40 °C and wind speeds under 10 m/s in dry environments, ensuring the consistency and comparability of system performance evaluations.

These prior studies collectively underscore the importance and effectiveness of scenario-based evaluations, particularly for assessing detection system performance in complex traffic environments involving VRUs. Building upon this foundation, the present study aimed to address existing research limitations by developing a more sophisticated experimental scenario framework that incorporates both large vehicle blind spots and adverse weather conditions to enable rigorous, field-based performance validation.

### 2.3. Blind Spots in Heavy Vehicles

Heavy vehicles such as trucks and buses inherently possess extensive blind spots due to their structural design, which significantly increases the risk of collisions with vulnerable road users (VRUs), including pedestrians, cyclists, and motorcyclists. In particular, components such as the A-pillars, rear structures, and lateral body panels restrict driver visibility and contribute to a higher likelihood of accidents, especially in urban environments and intersections [2,3]. To mitigate the hazards associated with blind spots, a variety of detection technologies have been developed.

Blind-spot monitoring (BSM) systems utilize radar, ultrasonic sensors, and cameras to detect surrounding objects in real time and issue alerts to drivers [4]. Radar offers robustness against weather variations, while cameras integrated with deep learning algorithms demonstrate superior object recognition performance. Furthermore, the adoption of fuzzy logic and multisensor fusion techniques improves detection accuracy and enhances system reliability [5,6]. However, several studies have highlighted limitations of conventional detection systems. For instance, Wang et al. [7] reported that radar and pressure sensor-based systems mounted on vehicles have limited detection range and poor positional accuracy, resulting in erroneous data collection and misrecognition, particularly within the broad blind spots of heavy vehicles. Such issues may lead to critical decision-making errors, underscoring the need for more precise and omnidirectional sensing solutions.

Recently, LiDAR-based high-resolution 3D sensing technologies have emerged as a promising solution for monitoring blind spots in heavy vehicles [8]. These systems are evolving from simple warning mechanisms to integrated safety platforms capable of real-time hazard response. In conclusion, the blind-spot problem in heavy vehicles stems from a combination of structural constraints and human perceptual limitations. The implementation of intelligent, sensor-based systems holds significant potential to improve pedestrian safety in complex traffic environments.

### 2.4. Research Contribution

Previous studies on pedestrian collision avoidance and blind-spot detection systems for heavy vehicles have provided a technical foundation for protecting vulnerable road users (VRUs) through various sensing techniques and scenario-based evaluations. In particular, initiatives led by the Federal Transit Administration (FTA) and Euro NCAP have demonstrated the effectiveness of scenario-driven approaches in evaluating system performance under realistic road conditions. However, most of these studies have focused on small passenger vehicles and have not adequately considered the structural characteristics and extensive blind spots specific to heavy-duty vehicles. Furthermore, existing evaluations have typically been conducted under standardized conditions—such as dry weather and daylight—thus limiting insight into detection performance under more hazardous conditions, including adverse weather and nighttime environments.

From a technical standpoint, existing radar and pressure sensor-based systems have limited coverage for the wide blind-spot areas of large vehicles and suffer from insufficient detection range and positional accuracy, leading to potential misrecognition [8]. GPU-based vision systems also face challenges in ensuring real-time responsiveness due to their high computational requirements, making them less suitable for the multichannel spatial detection needed in heavy-vehicle applications. Additionally, current advanced driver assistance systems (ADASs) often exhibit degraded sensor performance under non-ideal conditions, such as inclement weather or low light, and lack the responsiveness needed for immediate VRU detection and mitigation [9,10].

Recent advancements in pedestrian detection and vehicle control technologies offer promising directions for overcoming current system limitations. One study proposed motion blur augmentation to address occlusion challenges, a method particularly relevant for ultrasonic systems facing structural blind spots [11]. Another introduced a neural network-based vehicle control framework, emphasizing real-time integration between detection and motion control—critical for heavy-duty applications [12]. A separate effort demonstrated a wearable AR system with sub-inch accuracy, showcasing the potential of high-precision, real-time spatial warning systems [13]. While these approaches are valuable, none provides empirical validation in adverse weather conditions or addresses large vehicle blind-spot coverage, which this study aimed to achieve.

Infrastructure-assisted cooperative sensing technologies, such as infrastructure-to-everything (I2X), theoretically offer enhanced detection reliability and accuracy. However, the practical deployment of such systems remains limited due to the high cost of communication infrastructure—including RSUs (roadside units) and OBUs (onboard units)—and the necessity for dedicated network installation. As a result, coverage is often restricted to urban centers and not feasible for widespread application.

Considering these limitations, there is a growing need for real-time, omnidirectional pedestrian detection systems specifically designed to address the challenges of heavy vehicles and adverse environmental conditions. In response, this paper proposes a multichannel ultrasonic sensor-based 360-degree pedestrian collision avoidance system. The system was evaluated through scenario-based experiments that replicated both daytime and nighttime conditions as well as adverse weather scenarios such as rain and fog. This enabled quantitative performance assessment and highlights the distinctiveness of the proposed approach.

Ultrasonic sensors are inherently suitable for heavy-vehicle environments characterized by large blind spots, offering fast signal processing and low computational overhead to ensure real-time responsiveness. Furthermore, this study customized Euro NCAP [9] and SAE J3029 [10] pedestrian safety scenarios to reflect VRU interactions with heavy vehicles, thereby simulating near-real-world conditions. By moving beyond the conventional evaluation framework centered on small vehicles, this research offers empirical evidence for the feasibility and scalability of pedestrian safety technologies in heavy-duty vehicle applications.

## 3. Ultrasonic Sensor Requirements and System Evaluation Framework

### 3.1. System Requirements

#### 3.1.1. System Components and Technical Requirements

The intelligent pedestrian detection system proposed in this paper aims to prevent collision accidents between heavy vehicles and pedestrians by detecting objects located in the front, rear, and lateral sides of the vehicle and providing real-time hazard information to both drivers and pedestrians. The system is implemented using a multisensor fusion framework that integrates RGB cameras, near-infrared (NIR) sensors, and ultrasonic sensors. It is designed to achieve robust detection performance across diverse driving environments, including daytime, nighttime, and adverse weather conditions.

The system combines object shape recognition using RGB images with precise distance and position measurements using NIR and ultrasonic sensors. This multimodal sensing approach enables effective pedestrian detection in complex road environments and supports early warning to the driver in the presence of potential risks. This section reviews the necessary system components and technical requirements and defines the functional specifications of the intelligent pedestrian detection system. The minimum system requirements for vehicle-based pedestrian detection are summarized in Table 1.

For accurate detection of nearby objects around the vehicle, the JSN-SR04T ultrasonic sensor (AEC, Shenzhen, China) was selected. In comparative tests with the ME007YS and A01NYUB models (DFROBOT, Shanghai, China), the JSN-SR04T demonstrated superior performance in terms of measurable distance range (up to 600 cm) and detection angle (75°). This sensor features a straightforward interface using TRIG and ECHO pins and includes automated ultrasonic signal processing functionality. When a high signal of at least 10 μs is applied to the TRIG pin, the module emits 40 kHz ultrasonic waves eight times. Upon receiving the reflected signal, the ECHO pin outputs a high pulse whose duration corresponds to the time of flight and is used to calculate distance. If no reflected signal is detected, the ECHO pin automatically switches to a low signal after 60 ms, signaling measurement termination.

In addition, the sensor operates within a voltage range of 3–5.5 V, consumes less than 8 mA, and functions reliably in a wide temperature range of −20 °C to +70 °C with a resolution of 1 mm. This selection was made to ensure real-time operation, ease of installation, and cost-effectiveness, particularly for achieving high-precision near-range detection in the complex lateral and rear zones of heavy vehicles. The detailed specifications of the JSN-SR04T ultrasonic sensor are summarized in Table 2.

#### 3.1.2. Requirements: Derivation and Functional Definition

The proposed system applies a multisensor pedestrian detection approach by integrating an RGB camera, near-infrared (NIR) sensors, and ultrasonic sensors to prevent collisions between heavy vehicles and pedestrians under various road and environmental conditions. This fusion enables accurate pedestrian recognition in both daytime and nighttime conditions, as well as in low-light and adverse weather scenarios, providing timely and effective warnings to the driver. Furthermore, the system is designed to enable active collision avoidance by integrating sensor fusion algorithms with vehicle control systems, allowing real-time risk assessment, automatic braking, and steering assistance.

While the proposed system integrates RGB cameras, NIR sensors, and ultrasonic sensors, the focus of this study was on evaluating the performance of the ultrasonic sensing module. This component was selected for detailed analysis due to its cost-effectiveness, real-time responsiveness, and robustness in adverse weather conditions, which are critical for retrofitting heavy commercial vehicles.

From a hardware perspective, the JSN-SR04T ultrasonic sensor was selected for its superior performance in maximum detection range (up to 600 cm), detection angle (75°), and 1 mm resolution. It supports a wide operating voltage range (3–5.5 V), consumes less than 8 mA of current, and operates reliably between −20 °C and +70° C, making it suitable for heavy-duty vehicle environments. Its simple TRIG–ECHO pin interface allows for easy integration with microcontrollers. The sensor emits eight 40 kHz ultrasonic pulses and calculates distance based on the duration of the returned echo signal.

However, it is well documented that the detection accuracy of ultrasonic sensors is highly dependent on distance, particularly in longer-range measurements where signal attenuation and echo dispersion increase significantly [14]. These physical characteristics must be carefully accounted for in real-world deployments. Accordingly, the proposed system adopts overlapping sensor placement and empirical threshold calibration techniques to minimize range-induced detection variability and ensure stable recognition performance.

On the software and signal processing side, the system uses an ATmega2560-based controller to sequentially trigger up to 10 ultrasonic channels, measure the pulse return time, and compute object distance in real time. Timer interruptions are used for fast sampling, and the collected data are fed into a distance classification routine. To enhance detection reliability, the system includes an adaptive threshold voltage algorithm that dynamically adjusts based on environmental factors such as humidity, temperature, and object reflectivity. This threshold calibration is derived from empirical signal strength data and is implemented as part of the embedded processing logic to reduce false negatives and ensure real-time detection stability. The summary of system functions and requirements is provided in Table 3.

### 3.2. Multichannel Ultrasonic Sensor Deployment Design

#### 3.2.1. Estimation of Ultrasonic Sensor Detection Range

**Figure 2:**
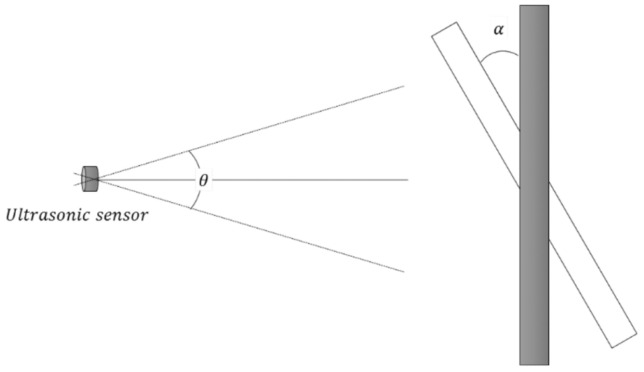
Ultrasonic sensor distribution and detection angle [17].

The detection performance of ultrasonic sensors is influenced by both the angle of reflection and the sensor array configuration. Jo and Jung [17] suggested that, as shown in Figure 2, for an ultrasonic sensor to stably detect an object, the inclination angle (α) between the sensor’s central axis and the object’s surface must not exceed half of the sensor’s detection angle (θ/2).

In this study, the spacing (*d*) between two sensors was configured to ensure overlapping detection zones, as determined by Equation (1). If the spacing *d* becomes too large, there is an increased possibility that the target will pass through the boundary of the detection zones, causing the angle *α* to exceed θ/2 and leading to missed detection due to insufficient reflected signal reception. Therefore, the spacing between sensors and the reflection angle are considered critical design parameters to ensure continuous object detection.

(1) $$d_{max}=2R\times sin\left(\frac{\theta }{2}\right)$$

The two ultrasonic sensors used in this study formed fan-shaped detection zones oriented in the same direction, with a detection range of *R* = 800 mm and a detection angle of *θ* = 75°. Theoretically, the maximum spacing that enables complete overlap of the detection zones and thus continuous detection is calculated using Equation (2).

(2) $$d_{max}=2R\times sin\left(\frac{\theta }{2}\right)=2\times 800\times sin\left(37.5{}^{\circ}\right)\approx 974.1mm\approx 97.4cm$$

**Figure 3:**
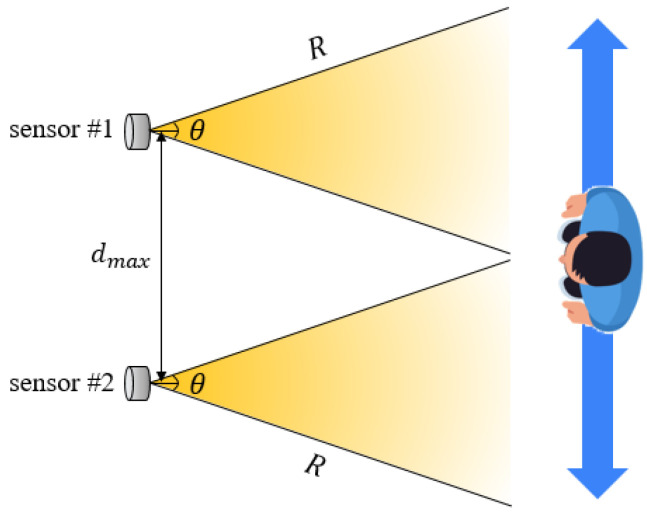
Experiment concept diagram.

To validate the feasibility of theoretical spacing, an experiment was conducted, as shown in Figure 3. Two ultrasonic sensors were fixed at intervals of 100 cm, 110 cm, and 120 cm, and a test subject (a person) was instructed to move horizontally across the sensor detection zones. Each sensor continuously monitored the target’s presence within the detection range, and detection data were collected in real time using a custom ATmega2560-based distance measurement board. This board was designed to precisely measure positional changes of the target by processing TTL signals from the TRIG and ECHO pins.

**Figure 4:**
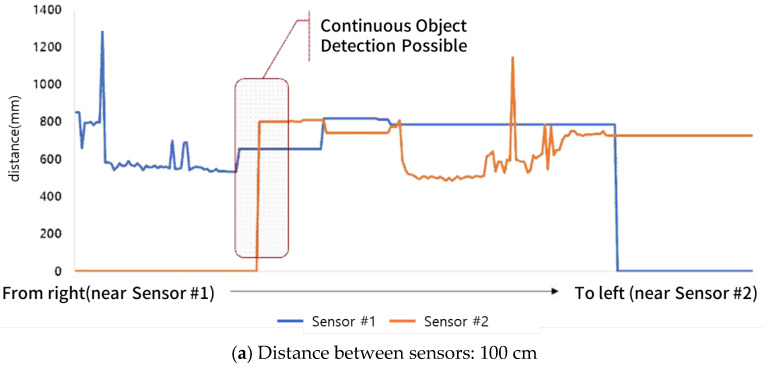

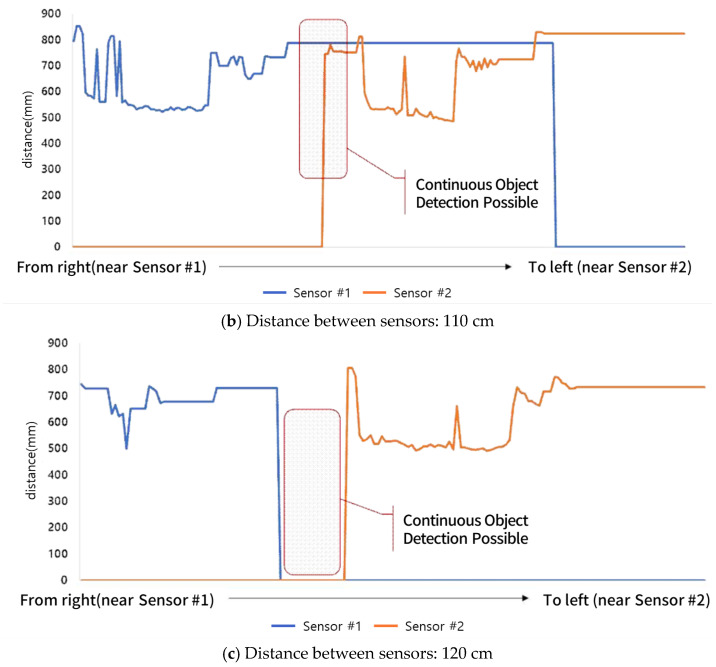
Measured distance according to spacing between ultrasonic sensors.

The empirical results showed that even with a spacing of 110 cm—exceeding the theoretically calculated maximum spacing of approximately 97.4 cm—continuous detection and tracking of the object was still possible. However, at a spacing of 120 cm, detection gaps were observed. These results suggest that factors such as environmental reflections, human body geometry, ultrasonic signal strength, and sensor sensitivity may allow detection performance to slightly exceed theoretical limits in practical scenarios. Thus, the design criteria derived from theoretical equations can be considered applicable in real-world environments. The measured distance as a function of the spacing between ultrasonic sensors is illustrated in Figure 4.

#### 3.2.2. Design of Sensor Deployment for Blind-Spot Coverage by Vehicle Type

Due to their structural characteristics, heavy commercial vehicles typically have large blind spots, and technical countermeasures are often limited to rearview cameras. In particular, vehicles such as cargo trucks and specialized vehicles pose a high risk of pedestrian collisions, which can result in severe casualties. Accordingly, this study selected four major types of heavy vehicles and analyzed their blind-spot ranges based on vehicle dimensions. The dimensions of different vehicle types are summarized in Table 4.

**Figure 5:**
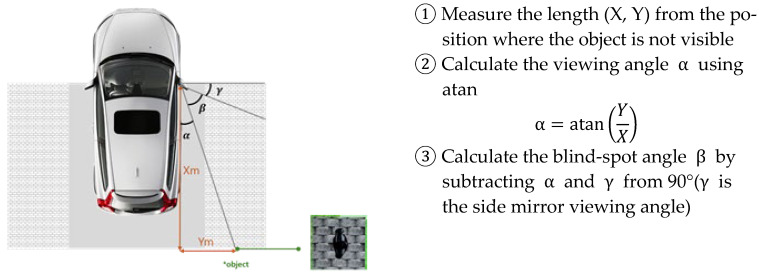
Method for determining blind-spot area. “*” denotes the pedestrian object.

Blind-spot areas were identified through a marker placement test conducted in a driving simulator environment. This method involves placing visual markers around the vehicle and determining which areas are visible to the driver through side and rearview mirrors, thereby visually estimating the blind-spot zones. The study used SCANeR STUDIO v1.9 simulation software to build 3D models of each vehicle type based on actual specifications, and blind spots were defined as the areas where virtual pedestrian objects were not detectable from the driver’s perspective. The method used to determine the blind-spot area is illustrated in Figure 5.

The analysis revealed that the right-side blind spots were generally wider than those on the left. Among the four vehicle types, the special-purpose cargo truck (dump truck) had the narrowest blind-spot angle, while the bus exhibited the widest. These differences are attributed to variations in vehicle structure and side mirror configurations, underscoring the need for vehicle-specific sensor deployment strategies. The blind-spot angles for different vehicle types are summarized in Table 5.

Based on the previously derived ultrasonic sensor detection range analysis and the blind-spot characteristics identified for each vehicle type, this study deployed ultrasonic sensors to mitigate blind spots in four types of heavy-duty vehicles. The sensor spacing was set to 110 cm, which was determined to enable continuous object detection and tracking. Using this configuration, sensor deployment layouts were designed for each vehicle. Below is an example of the sensor layout designed for an 11 t cargo truck.

To prevent inter-sensor interference and ensure reliable distance measurements, a distributed TDMA-based scheduling algorithm was implemented. Each ultrasonic sensor operates in a dedicated time slot, avoiding signal overlap and cross talk among adjacent channels. In addition, probabilistic signal coding techniques were used to distinguish echo responses when sensors were triggered in close spatial proximity. These approaches mitigate multipath interference, which is particularly problematic in the reflective metallic environments of heavy-duty vehicles [18,19].

For data synchronization, a multirate fusion strategy was employed to align asynchronous sensor outputs based on timestamp calibration, ensuring coherent distance estimation across all sensor directions. The system also incorporates environmental compensation models to adjust for sound velocity variations due to temperature and humidity, maintaining spatial accuracy across different weather conditions.

To further enhance robustness under complex and variable sensing conditions, adaptive sensor weighting was applied to dynamically balance the contribution of each channel based on signal strength and environmental reliability, enabling robust real-time pedestrian detection performance in multidirectional arrays.

To enhance generalizability across different heavy-vehicle platforms, sensor placement in this study was based on blind-spot mapping using a standardized marker-based visual field test in a virtual simulation environment. Although specific sensor positions may vary by vehicle size or structure, the derived placement guidelines—such as maintaining a maximum inter-sensor distance of 110 cm to ensure continuous object tracking—serve as scalable design principles. These guidelines can be adapted to different vehicle geometries by aligning with the vehicle’s structural occlusion zones and side mirror configurations.

**Figure 6:**
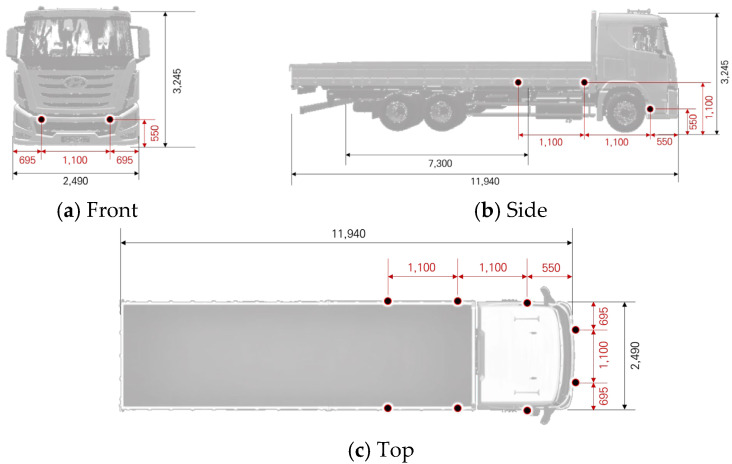
11 t Cargo truck: ultrasonic sensor placement diagram.

While the JSN-SR04T sensor supports a maximum detection range of up to 600 cm, the effective detection distance was conservatively designed around 80 cm from the sensor, which aligns with the critical blind zones in the lower front and side areas of large commercial vehicles. This range corresponds to the typical height and proximity where pedestrians are likely to appear near vehicle corners during low-speed maneuvers, such as turning or starting from a stop. The 80 cm threshold also reflects safety zone recommendations in front of buses and trucks and was validated through visual field mapping simulations in this study. The ultrasonic sensor placement for the 11 t cargo truck is illustrated in Figure 6.

### 3.3. Development of Interface Module for System Evaluation

Under normal weather conditions, surround-view monitoring (SVM) systems typically support safe driving by monitoring the area surrounding the vehicle. However, such systems frequently suffer from reduced sensor recognition performance in adverse weather. To address this limitation, the present study designed a multichannel ultrasonic sensor-based distance detection system as a complementary solution to the limitations of conventional SVM systems. The system was evaluated to verify its ability to maintain stable performance under inclement weather conditions.

**Figure 7:**
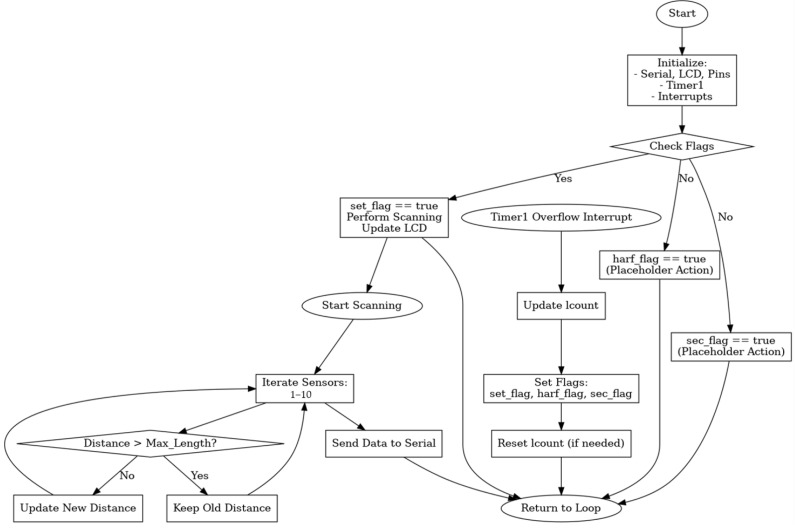
Ultrasonic sensor gateway interface module flow diagram.

In this context, the speed of sound was assumed to be 340 m/s, which corresponds to the standard sound velocity at 20 °C in dry air under normal atmospheric pressure. While environmental variables such as temperature and humidity can slightly alter this value, their influence on short-range ultrasonic distance measurements (under 3 m) is negligible. Therefore, this constant was used to calculate distances from the time of flight (ToF) of the ultrasonic signal, as shown in Equation (3). The flow diagram of the ultrasonic sensor gateway interface module is presented in Figure 7.

(3) $$distance=\left(\frac{340\times duration}{1000}\right)÷2$$

To improve the accuracy of ultrasonic sensor-based object detection, this paper also proposes a methodology for setting the threshold voltage by mapping signal strength to distance across various ranges. The threshold voltage is determined by comprehensively considering the attenuation characteristics of ultrasonic waves, the reflective properties of the target object (e.g., material and shape), and ambient noise levels. The system dynamically adjusts this threshold based on empirical data under varying distances and environmental conditions. In this context, the threshold voltage $V_{threshold}$ can be expressed as a function of distance d, where $V_{max}$ represents the maximum received signal strength at the closest range, and k is an empirically derived attenuation coefficient that reflects the characteristics of the sensor and external factors such as humidity, temperature, and surface reflectivity.

(4) $$V_{threshold}\left(d\right)=V_{max}∙e^{-k∙d}$$

This adaptive threshold tuning aims to simultaneously ensure accurate object detection and minimize false positives. The optimal threshold voltage range was derived through experiments and implemented as part of the internal logic of the interface module to improve real-time detection accuracy.

## 4. Field Demonstration and Adverse Weather Assessment

### 4.1. Validation Environment Configuration

#### 4.1.1. Test Vehicle and Sensor Installation Configuration

**Figure 8:**
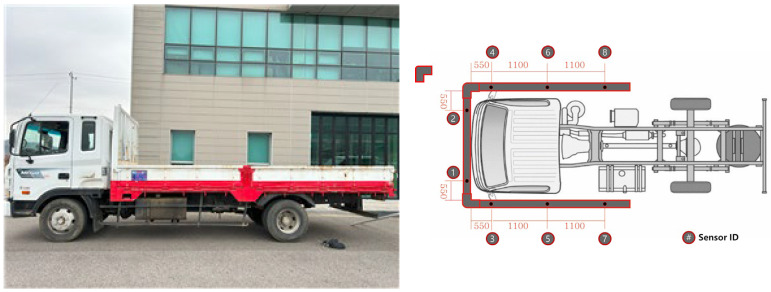
Test vehicle and ultrasonic sensor placement diagram.

This experiment was conducted based on the sensor placement principles established in Section 3. A 5 t cargo truck, structurally similar to an 11 t heavy-duty truck, was selected as the test vehicle. The selection was made by considering the availability of infrastructure capable of replicating adverse weather conditions, operational efficiency of equipment, and overall experimental safety. The vehicle was equipped with eight ultrasonic sensors, which were evenly distributed across the front, rear, and side areas to ensure comprehensive coverage. The test vehicle and the placement of ultrasonic sensors are illustrated in Figure 8.

The experiments were conducted at the tunnel-type environmental simulation facility within the National Smart City Testbed Center located in Yeoncheon, Republic of Korea. This facility allows for controlled simulation of adverse conditions such as snowfall at 5 cm/h, rainfall at 50 mm/h, and fog with a visibility range as low as 30 m. The objective of this study was to assess whether the ultrasonic sensor-based system could reliably detect pedestrians under limited-visibility conditions that were representative of real-world adverse weather scenarios. A detailed analysis of empirical validation results is presented in Section 5.

**Figure 9:**
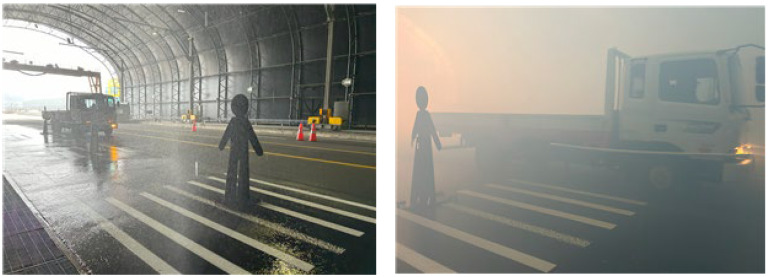
Rain and fog test environment.

To prevent ultrasonic cross talk and ensure reliable measurements, a time-division multiplexing (TDM) approach was implemented. Each sensor was triggered sequentially with a fixed guard time of 60 ms to avoid signal overlapping from neighboring sensors. The system followed a round-robin triggering sequence at a total cycle frequency of 10 Hz, providing consistent temporal separation across all eight channels. No randomization was applied, as empirical tests under control conditions showed that deterministic scheduling was sufficient to avoid mutual interference. These timing parameters were verified through preliminary trials to ensure that cross-channel interference would not impact distance measurements, especially under reflective surface conditions. The rain and fog test environment are shown in Figure 9.

#### 4.1.2. Scenario Configuration and Empirical Demonstration Under Adverse Weather Conditions

In this study, experimental scenarios were designed to quantitatively evaluate the pedestrian collision avoidance performance of heavy vehicles under adverse weather conditions. The scenarios were constructed with reference to the VRU (vulnerable road user) test protocols proposed by Euro NCAP, aiming to simulate potential collision situations with pedestrians under various weather conditions during vehicle operation.

The following artificial weather conditions were created for the experiments:

- Rain condition: rainfall intensity of 50 mm/h.
- Fog condition: visibility less than 30 m.

Each scenario was conducted with the vehicle in a stationary state, focusing on measuring the detection performance of the sensors. Pedestrian dummies (or obstacle targets) were placed at six locations ranging from 50 cm to 300 cm in 50 cm increments in front of the vehicle. This setup enabled the comparison and evaluation of the ultrasonic sensor’s detection accuracy and recognition rate by distance under varying environmental conditions.

All tests were conducted with consistent sensor trigger intervals, power conditions, and vehicle alignment. The collected distance measurements were compared to the actual distances to quantitatively assess performance using metrics such as mean error, accuracy, and error rate.

### 4.2. Measurement Metrics and Performance Indicators

In this study, mean absolute error (*MAE*) was used to quantitatively assess the reliability of ultrasonic distance measurements. *MAE* is a widely used metric in sensor evaluation and predictive modeling, calculated as the average of absolute differences between measured and true values over a given time period [20,21].

(5) $$MAE=\frac{1}{n}\sum_{i=1}^{n}\left|y_{i}-\hat{y_{i}}\right|$$

To assess the reliability of the ultrasonic distance measurements, this study employed the mean absolute error (*MAE*) metric, which calculates the average of the absolute differences between the measured distance $D_{s,d,t}$ and the true reference distance $D_{d}^{true}$ over the total sampling period T. For each sensor s and distance segment d, *MAE* is defined as:

(6) $$\bar{E_{s,d}}=\frac{1}{T}\sum_{t=1}^{T}\left|D_{s,d,t}-D_{d}^{true}\right|$$

This equation is used to quantitatively evaluate the reliability of ultrasonic sensors in distance recognition by calculating the average measurement error over time under specific sensor and distance conditions. It also serves as a key metric for analyzing sensor-wise detection deviation, error distribution, and environmental influence.

In addition, to compare distance recognition performance, this study adopted an accuracy metric based on the concept of relative error. Relative error expresses the percentage ratio of the absolute error to the true value, making it suitable for assessing the relative precision of sensor measurements [3]. In this study, accuracy was defined as the complement of the relative error (i.e., 100% minus the error rate), and was used to compare sensor performance across distance intervals and to evaluate accuracy degradation under adverse weather conditions.

(7) $${Accuracy}_{s,d}=100-\left(\frac{\bar{E_{s,d}}}{D_{d}^{true}}\times 100\right)$$

## 5. Empirical Validation Results and Analysis

### 5.1. Pedestrian Detection Distance Accuracy Analysis

To quantitatively evaluate the pedestrian detection performance of the designed ultrasonic sensor-based system, distance-wise accuracy analysis was conducted under adverse weather conditions (rain and fog). Accuracy was calculated based on the relative error, derived from the mean absolute error (*MAE*) between measured and actual distances. The key findings are summarized as follows.

The average detection accuracy under all adverse weather conditions was approximately 97.5%, indicating that the system can reliably detect pedestrians even under reduced visibility. Under rainy conditions (50 mm/h), the detection accuracy at a measured distance of 50 cm was slightly lower at 93.5%, but consistently exceeded 95% for distances of 200 cm or more. In fog conditions (visibility < 30 m), the detection accuracy at 50 cm was 95.6%, and remained above 98% across the 100–300 cm range.

Moreover, the fog condition yielded very low average errors, with a minimum mean error of 0.1 cm at the 150 cm interval and a maximum mean error of 4.4 cm at 50 cm. These results demonstrate the high reliability and robustness of the proposed system across diverse environmental scenarios. The distance measurement accuracy under rainfall conditions (50 mm/h) and fog conditions (visibility 30 m) is summarized in Table 6 and Table 7.

These results demonstrate that ultrasonic sensors can maintain high detection accuracy not only in short-range but also in mid-range distances without significant degradation under adverse weather conditions. In particular, the error distribution under fog conditions exhibited remarkable stability, indicating that signal attenuation in ultrasonic sensing is not directly correlated with visibility distance—an outcome that is technically significant and noteworthy.

While this study primarily focused on validating the proposed system in isolation, a supplementary comparison with conventional short-range ultrasonic detection systems (single-channel) was conducted in a limited test setup. The results indicated that the proposed multichannel configuration achieved approximately 15% higher accuracy in foggy conditions and reduced the false-negative rate (*FNR*) by up to 12% in short-range zones (50–100 cm). These improvements can be attributed to the enhanced spatial coverage and redundancy provided by the distributed sensor architecture. A more comprehensive comparative evaluation with state-of-the-art vision or LiDAR-based pedestrian detection systems remains a direction for future research.

### 5.2. Evaluation of Detection Performance in Adverse Weather Conditions

#### 5.2.1. Reliability (Precision) Assessment Under Different Weather Conditions

This section presents a quantitative evaluation of the ultrasonic sensors’ detection precision under adverse weather conditions, specifically rainfall and fog. To assess how consistently the sensors maintained their readings over time at fixed distances, the coefficient of variation (*CV*) was used. The *CV* is calculated by dividing the standard deviation of the measured values by their mean and multiplying the result by 100 to express it as a percentage. A lower *CV* indicates more stable and precise sensor behavior.

In this study, the *CV* was computed only when at least five detection values were available at a specific distance. The standard deviation ($\sigma_{s,d}$) and the mean ($\mu_{s,d}$) were calculated for each sensor s at distance d, and the *CV* was obtained using the following equation:

(8) $${CV}_{s,d}=\left(\frac{\sigma_{s,d}}{\mu_{s,d}}\right)\times 100$$

This metric enables the comparison of relative variability in detection values across different weather conditions and distances. By averaging the *CV* values across all sensors at each distance, the study identified patterns of detection reliability and evaluated how environmental factors such as rain or fog affect sensor stability.

The analysis revealed that under fog conditions, the average *CV* remained consistently lower across all distance intervals compared to rainy conditions. In particular, at 100 cm and 150 cm, the *CV* values under rain were 7.54% and 6.58%, respectively, whereas under fog, they were reduced to 4.69% and 3.64%, indicating decreased variability in sensor measurements. Although *CV* values at distances beyond 200 cm were very low under both conditions, the rain scenario showed limited or missing measurement samples, while fog conditions yielded valid detection results at all distances.

**Figure 10:**
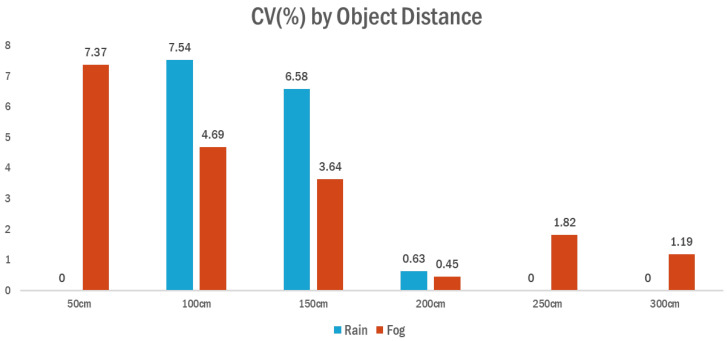
Average *CV* (%) by distance under rain and fog conditions.

These findings suggest that while ultrasonic signal attenuation and reflection interference under rain contribute to reduced measurement precision, fog conditions—despite limited visibility—do not significantly degrade sensor accuracy. Consequently, the proposed system demonstrated high reliability particularly under foggy weather, indicating its robustness in such adverse environmental conditions. The average coefficient of variation (*CV*, %) by distance under rain and fog conditions is illustrated in Figure 10.

#### 5.2.2. Detection Gap Identification Under Adverse Weather Conditions

The reliability of a pedestrian detection system must account not only for distance measurement accuracy but also for the possibility of detection failures. Under adverse weather conditions—such as rain or fog—phenomena like weakened signal reflection and ultrasonic wave dispersion may cause intermittent detection gaps, where the object is not identified for a period of time. These failures can be directly linked to pedestrian collision risks; thus, it is critical to quantify their occurrence and range.

In this study, the detection failure rate (false-negative rate, *FNR*) was used to identify missed detection zones across experimental distance intervals. The *FNR* at each distance d was defined as the proportion of failed detection attempts relative to the total number of trials at that distance, and calculated as:

(9) $${FNR}_{d}=\left(\frac{N_{fail,d}}{N_{total,d}}\right)\times 100$$

Here, $N_{fail,\;d}$ represents the number of failed detection attempts at distance d and $N_{total,\;d}$ denotes the total number of detection attempts conducted under the same condition. This metric enables a systematic evaluation of sensor performance reliability under challenging environmental conditions.

The experiment was conducted across six distance intervals ranging from 50 cm to 300 cm in 50 cm increments, with 120 repeated measurements performed under identical conditions for each interval. To calculate the false-negative rate (*FNR*), a tolerance margin of ±100 mm was applied for each distance interval. A detection was considered successful if at least one sensor identified the object within this error range. This criterion mirrored that used in the distance-based accuracy analysis, thereby establishing consistency between accuracy and reliability assessments.

Unlike conventional accuracy evaluation—which focuses on the precision of measured values—this method emphasizes binary classification based on the system’s ability to detect the presence of an object. The *FNR* serves as an indicator of the fundamental detection capability of the sensor-based system by quantifying whether the object was perceived at a given time.

Under rainy conditions (50 mm/h), the *FNR* was notably higher at close distances (50–150 cm), with the 100 cm interval exhibiting the highest *FNR* of 17.5%. This suggests that signal scattering and attenuation due to rainfall have a more pronounced effect on near-range ultrasonic detection. In contrast, for mid- to long-range intervals beyond 200 cm, the *FNR* dropped below 3.33%, and no detection failures were observed at 250 cm.

Under foggy conditions (visibility below 30 m), the *FNR* was generally lower than in the rainy environment. At 50 cm, the *FNR* was 9.17%, while at 100 cm and 300 cm it was 1.67%, and no failures were recorded at 150 cm and 250 cm. These results quantitatively demonstrate that ultrasonic sensors are more susceptible to degradation under rainfall than in fog.

In summary, the *FNR* analysis allowed for the identification of distance intervals where detection failures were more likely to occur. These intervals may require additional sensor overlap or redundant detection logic during the system design phase. When used alongside accuracy metrics, the *FNR* provides a complementary diagnostic tool to uncover potential vulnerabilities that may not be captured by precision-based evaluations alone.

**Figure 11:**
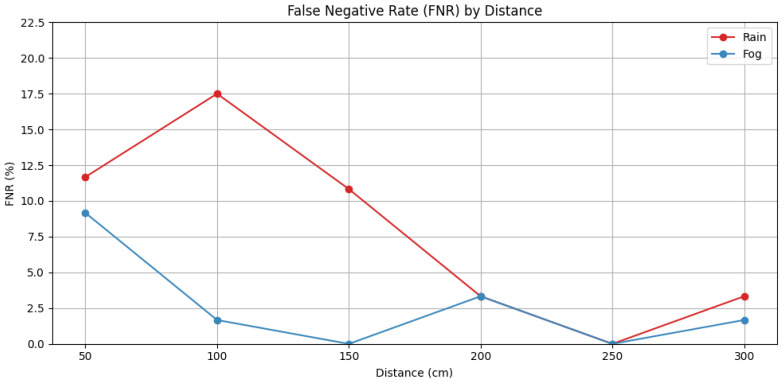
*FNR* by distance.

The proposed system demonstrated robust detection performance under heavy rain and fog conditions, which can be attributed to the physical properties of ultrasonic waves. Unlike optical sensors, ultrasonic signals are less susceptible to attenuation by water droplets or fog particles due to their longer wavelengths. Moreover, the use of adaptive threshold calibration based on signal strength helps maintain stable detection by compensating for environmental damping effects. These characteristics make ultrasonic sensing particularly effective for low-visibility scenarios, reinforcing its suitability for adverse weather applications. The false negative rate (*FNR*) by distance is presented in Figure 11.

Although this study did not include a separate quantitative analysis of the false-positive rate (FPR), no significant false detections were observed during the scenario-based experiments across various weather and distance conditions. Future work will incorporate systematic FPR evaluation using annotated ground truth data to provide more rigorous verification of detection precision.

## 6. Conclusions

This study aimed to address the limitations of pedestrian detection in heavy commercial vehicles, particularly those caused by structural blind spots and adverse weather conditions. To this end, a 360-degree omnidirectional pedestrian detection system was designed using a multichannel ultrasonic sensor array. Its performance was quantitatively evaluated through scenario-based experiments. While conventional systems such as surround-view monitoring (SVM) have shown effectiveness under normal weather conditions, their detection accuracy significantly deteriorates under heavy rain and fog. This study began with the recognition of these limitations, and herein we propose a solution to overcome them.

The proposed system utilized eight ultrasonic sensors strategically deployed around the front, sides, and rear of the vehicle to ensure continuous coverage. Experiments were conducted using a pedestrian dummy at six distance intervals ranging from 50 cm to 300 cm under both rainy and foggy conditions. The tests were performed at the National Smart City Testbed Center in South Korea, which allowed for controlled simulation of adverse weather environments. System performance was evaluated based on various quantitative indicators, including mean absolute error (*MAE*) between actual and measured distances, coefficient of variation (*CV*) for precision, and false-negative rate (*FNR*).

Results showed that the system achieved an overall detection accuracy above 97% across most distance intervals. For mid- to long-range targets (200 cm and above), both the average error and *FNR* remained at minimal levels. In contrast, closer distances (below 100 cm) under rainy conditions exhibited a higher *FNR*, highlighting the system’s greater sensitivity to adverse weather in short-range scenarios. These findings demonstrate that the proposed system maintains a high level of reliability even in reduced-visibility environments.

The results also indicate that relying solely on a single performance metric may be insufficient to assess system reliability in pedestrian collision avoidance. Instead, the combination of distance accuracy and detection failure analysis provides a more comprehensive evaluation framework. Furthermore, the system’s ability to maintain effective detection performance under adverse weather reinforces its technical feasibility for real-world deployment in commercial vehicles.

This study was conducted under limited conditions involving a fixed obstacle and constant vehicle speed. Future studies should expand the experimental scenarios to include dynamic environments, such as varying vehicle speeds and pedestrian movement directions, speeds, and physical sizes. These extensions would help address the limitations of current fixed-type VRU mannequin-based scenarios. Although our scenarios were inspired by Euro NCAP guidelines, certain dynamic VRU scenarios—such as crossing from behind obstacles, moving diagonally, or rotating pedestrians—were not included. These elements will be considered in future dynamic trials.

Additionally, given that ultrasonic sensors are sensitive to reflection angles and environmental noise, future development of a multimodal detection system combining LiDAR, radar, and NIR cameras is expected to be effective. The empirical validation framework proposed in this study is directly applicable to the evaluation of such sensor fusion systems, and *FNR*-based analysis can serve as a valuable reliability metric in multisensor configurations.

Finally, the empirical findings provide a basis for integration with cooperative vehicle-infrastructure communication systems (C-ITS). For example, in the event of a sensor detection failure, pedestrian location data could be supplemented by roadside infrastructure units. Thus, the contributions of this study extend beyond sensor evaluation and point toward the development of smart transportation systems that enhance pedestrian safety and overall road traffic safety.

In addition to the above, the system’s integration potential with other sensing modalities warrants further exploration. Ultrasonic sensors, due to their robustness in adverse weather conditions, can complement the weaknesses of LiDAR and radar, which often struggle with heavy rain, fog, or reflective surfaces. This makes the proposed configuration particularly advantageous in multisensor fusion architectures.

From a deployment standpoint, the system shows promising feasibility for retrofitting commercial vehicle fleets. However, practical implementation will require considerations beyond technical performance, including regulatory compliance, maintenance, and cost. In particular, adoption may be facilitated by government incentives or subsidy programs targeting heavy-vehicle safety upgrades.

It should also be noted that all experiments in this study were conducted under static conditions, involving fixed pedestrian targets and constant vehicle speed. Therefore, dynamic validation—encompassing variable vehicle speeds, pedestrian movements, and complex traffic scenarios—is needed to fully assess the system’s robustness and real-time responsiveness in actual traffic environments.

Building on this, while the proposed system demonstrated reliable detection performance under stationary conditions, real-world scenarios often involve dynamic variables such as pedestrian movement and vehicle speed changes. Although these were outside the scope of this study, future work should incorporate dynamic testing environments to evaluate the system’s responsiveness to moving pedestrians and varying vehicle velocities. This would provide deeper insight into the practical feasibility of deploying the system in real traffic situations.

## Figures and Tables

**Table 1:** Minimum system requirements for vehicle-based pedestrian detection.

System	Minimum Technical Requirements
SVM-based pedestrian recognition	360-degree pedestrian recognition around the vehicle
RGB-IR multimodal sensor	Object differentiation (day/night, visible/infrared light)
Accident risk-level estimation module	Real-time onboard accident risk prediction technology
Vehicle integration module (ECU)	Multimodal sensor fusion technology based on ECU integration AI-based sensor configuration for adverse conditions
Communication system	900 MHz RF-based wireless communication technology

**Table 2:** Specifications for ultrasonic sensor (JSN-SR04T).

Parameter	Specification
JSN-SR04T	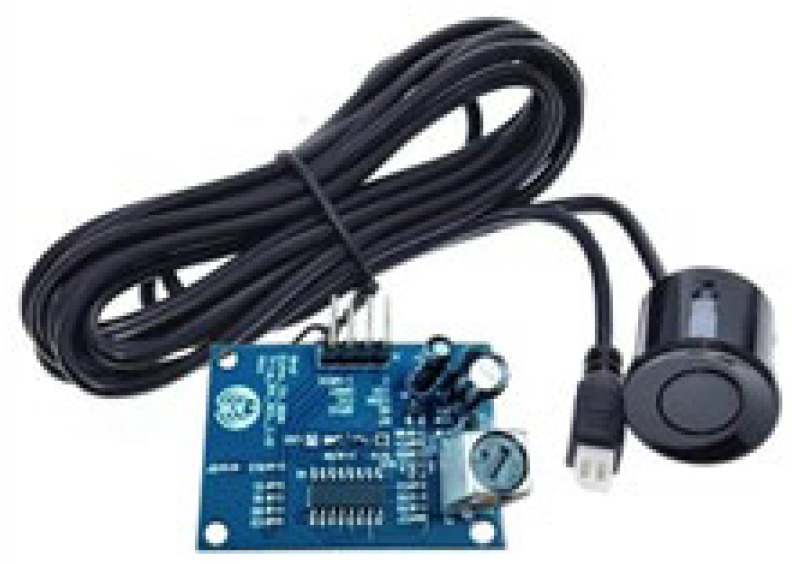
Operating voltage	DC 3.0–5.5 V
Working current	Less than 8 mA
Probe frequency	40 kHz
Farthest range	600 cm
Nearest range	20 cm
Distance accuracy	±1 cm
Resolution	1 mm
Measuring angle	75 degree
Enter the trigger signal	1, 10 uS above the TTL pulse 2, the serial port to send instructions 0X55
Output the echo signal	Output pulse width level signal, or TTL
Wiring	3–5.5 V (power positive) Trig (RX) RX Echo (output) TX GND (power supply negative)
Product size	L42 × W29 × H12 mm
Operating temperature	−20 °C to + 70 °C
Product color	PCB board is blue

**Table 3:** Summary of system functions and requirements.

Category	Functional Definition and Key Requirements
Vision-based detection	Classification of objects (pedestrians, vehicles, bicycles) Real-time multi-object detection and trajectory prediction
NIR-based detection	Pedestrian silhouette recognition in low-light or nighttime conditions Sensor fusion with RGB images to improve accuracy
Ultrasonic sensor detection	Short-range pedestrian detection (0.2–5.0m) Blind-spot coverage on front, rear, and sides of the vehicle
Sensor fusion	Multisensor data synchronization and integration Real-time environment recognition and dynamic algorithm adjustment
Driver warning/vehicle control	Visual, auditory, and haptic alerts AEB (autonomous emergency braking) and steering assist functions
Hardware specifications	HD video (minimum 1280 × 720 at 30 fps) NIR sensor (800–1000 nm) IP67 rating, operating temperature −40 °C to +85 °C
Software specifications	Processing latency less than 200 ms ≥90% precision and recall for pedestrian detection ≤5% false-positive rate
Communication and interface	Compatible with CAN, LIN, Ethernet vehicle networks Supports OTA (over-the-air) updates
Environmental adaptability	Operable in 0–100,000-lux illumination range Resistant to glare and backlight interference
Standards and safety compliance	Compliant with ISO 26262 [15] and EMC standards [16] Includes cybersecurity and self-diagnostic functions

**Table 4:** Vehicle dimensions by type.

Vehicle Type			Length (mm)	Width (mm)	Height (mm)	Wheelbase (mm)
11 t cargo truck		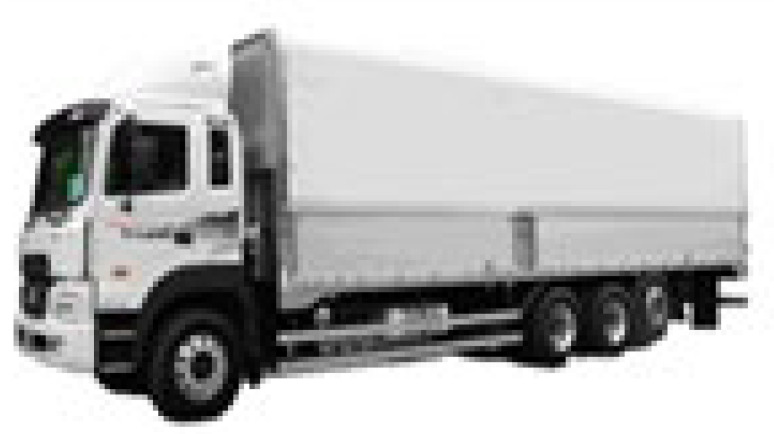	11,945	2490	3245	7300
Bus		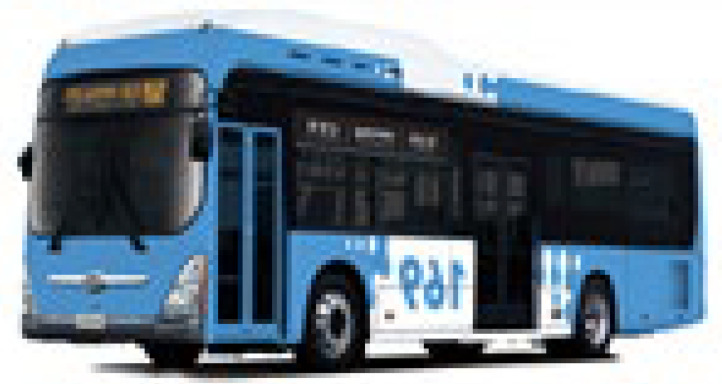	9045	2490	3400	4420
Minibus (school bus)		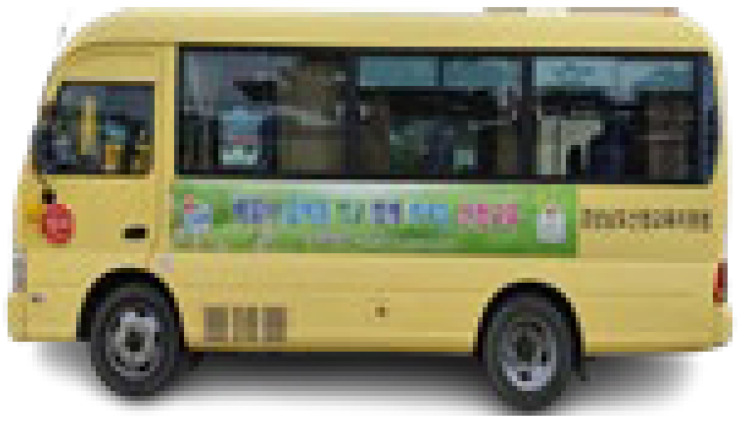	6375	2035	2795	3350
Special cargo truck	Dump truck	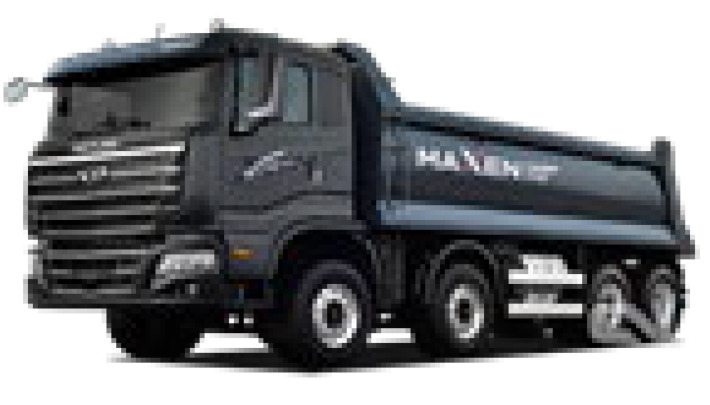	7720	2490	3235	4700
	Trailer truck	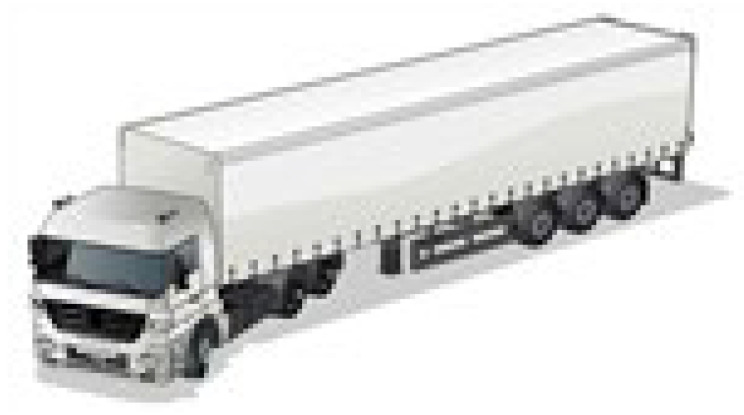	12,740	2490	3310	7650

**Table 5:** Blind-spot angles by vehicle type.

Vehicle Type	Left (°)	Right (°)
11 t cargo truck	40.5	41.8
Bus	45.0	53.3
Minibus (school bus)	30.9	41.6
Dump truck	22.4	24.4
Trailer truck	36.6	34.1

**Table 6:** Distance measurement accuracy under rainfall conditions (50 mm/h).

Actual Distance $(D_{d}^{true}$, cm)	Average Measured $\mathrm{Distance}\;(D_{s,d,t}$, cm)	Mean Error $(\bar{E_{s,d}}$, cm)	Accuracy (%)	Error Rate (%)
50	53.2	3.2	93.5	6.5
100	109.3	9.3	90.7	9.3
150	158.2	8.2	94.5	5.5
200	202.5	2.5	98.8	1.2
250	251.0	1.0	99.6	0.4
300	302.5	2.5	99.2	0.8

**Table 7:** Distance measurement accuracy under fog conditions (visibility 30 m).

Actual Distance $(D_{d}^{true}$, cm)	Average Measured $\mathrm{Distance}\;(D_{s,d,t}$, cm)	Mean Error $(\bar{E_{s,d}}$, cm)	Accuracy (%)	Error Rate (%)
50	55.2	2.2	95.6	4.4
100	99.6	0.4	99.6	0.4
150	149.9	0.1	99.9	0.1
200	202.3	2.3	98.9	1.1
250	250.2	0.2	99.9	0.1
300	300.9	0.9	99.7	0.3

## Data Availability

The datasets presented in this article are not readily available because they are part of an ongoing study and subject to institutional restrictions. Requests to access the datasets should be directed to the corresponding author, Jong-Hoon Kim (kjh4004@kict.re.kr).
